# Association of Rheumatoid Arthritis With Progression of Cognitive Impairment and Risk of Mortality in People With Dementia

**DOI:** 10.1212/WNL.0000000000213405

**Published:** 2025-02-07

**Authors:** Minjia Mo, Maria Eriksdotter, Sofia Ajeganova, Sumonto Mitra, Sara Garcia-Ptacek, Hong Xu

**Affiliations:** 1 Division of Clinical Geriatrics, Department of Neurobiology, Care Sciences and Society, Karolinska Institutet, Stockholm, Sweden;; 2 Theme Inflammation and Aging, Karolinska University Hospital, Stockholm, Sweden;; 3 Department of Medicine Huddinge, Karolinska Institutet, Stockholm, Sweden; and; 4 Rheumatology Division, Department of Clinical Sciences, Universitair Ziekenhuis Brussel, Vrije Universiteit Brussel, Belgium.

## Abstract

**Background and Objectives:**

Rheumatoid arthritis (RA) has been linked to an increased risk of dementia, yet little is known about how RA affects the progression of cognitive impairment and the risk of mortality in people with dementia. We aimed to investigate whether RA is linked to an accelerated cognitive decline and a higher risk of all-cause mortality in patients with dementia.

**Methods:**

We conducted a propensity score-matched register-based cohort study based on the Swedish Registry for Cognitive/Dementia Disorders-SveDem. Patients diagnosed with dementia and registered in SveDem between May 1, 2007, and October 16, 2018, were included. The main outcome for the study was cognitive decline, measured by Mini-Mental State Examination (MMSE) score changes over years. The secondary outcome was all-cause death. We used mixed-effects models to examine the association between RA and cognitive decline, and Cox proportional hazards models to investigate the risk of all-cause mortality. We also conducted subgroup analyses to explore the potential effects of sociodemographic, baseline MMSE, comorbidities, and the use of dementia medications on the association between RA and outcomes.

**Results:**

We included 1,685 dementia patients with RA (mean [SD] age, 79.9 [6.7] years; 73.4% were women) and 5,055 dementia patients with non-RA (80.1 [7.5] years; 73.1% were women). The median follow-up was 2.9 years (interquartile range, 1.5–4.6 years) for non-RA and 2.6 years (interquartile range, 1.4–4.2 years) for RA. In total, 111,266 MMSE measurements were available for analysis. Compared with non-RA patients, patients with RA presented faster cognitive decline (β = −0.24 points/y; 95% CI −0.38 to −0.10) and an increased risk of death (hazard ratio 1.15; 95% CI 1.06–1.24). In subgroup analysis, significant interactions were observed between RA and baseline MMSE scores as well as living conditions regarding cognitive decline (*p* for interaction <0.05).

**Discussion:**

We identified a worse cognitive function and an increased mortality risk in dementia patients with RA compared with non-RA. However, we lacked information on the duration of RA before the onset of dementia and on disease activity, which could influence our findings. Further studies are needed to validate these results in comparable populations.

## Introduction

Dementia is a significant contributor to mortality for individuals aged older than 70 years and accounts for 4.4% of total deaths.^1^ In Sweden, a high-income country with an aging population, an estimated 150,000 individuals suffer from dementia, two-thirds of whom have Alzheimer disease (AD).^2^ Increased longevity has led to a growing prevalence of dementia, associated with increased social and health care burden, various health problems, higher mortality risk, and shortened life expectancy in aged population.^3^ Current approved treatments for AD in Europe (donepezil, galantamine, rivastigmine, and memantine) are only symptomatic and have modest benefits.^4^ More recently, aducanumab^5^ and lecanemab^6^ effectively remove β-amyloid from the brain and reduce cognitive and functional decline in people living with early AD, but these are not approved in Europe. However, these drugs come with adverse events, and their long-term efficacy and safety remain unclear.^6,7^

AD involves intricate neuropathologic mechanisms, including the accumulation of extracellular Aβ plaques and the formation of intracellular neurofibrillary tangle with phosphorylated tau protein.^8^ Neuroinflammation is increasingly recognized as a potential target for AD.^9^ Inflammatory markers in CSF, plasma, and postmortem brain tissue of patients with AD underscore the significance of inflammatory process in AD.^8,9^ Furthermore, most cases of dementia are of mixed etiology, encompassing both vascular and neurodegenerative components. Inflammation has also been implicated in the pathogenesis of other types of dementia, such as vascular dementia (VaD).^10^ Addressing the pressing need for alternative therapeutic strategies in dementia, it is critical to identify inflammatory risk factors associated with dementia to develop therapeutics that can potentially delay or reverse age-related cognitive decline.

Rheumatoid arthritis (RA) is a systemic autoimmune disease characterized by chronic polyarticular inflammation and destruction of cartilage and bone structure.^11^ The age-standardized prevalence and incidence rates of RA are increasing globally,^12^ with an estimated prevalence of 0.51% in Sweden, similar to other European countries.^12^ RA is associated with chronic inflammation, leading to systemic complications such as cardiovascular disease, pulmonary disease, and dementia.^13,14^ In addition, there are shared genetic features between RA and AD,^15^ but the interaction has not been clarified. While some studies do not support a clear (causal) relation between RA and dementia,^16,17^ it should be noted that the use of disease-modifying antirheumatic drugs (DMARDs) in rheumatic patients may reduce the risk of developing dementia.^13,18^ RA is commonly treated with conventional DMARDs (csDMARDs), in some patients in combination with biologic DMARDs, targeted synthetic DMARDs (tsDMARDs), and glucocorticoids.^19^ The use of combination antirheumatic therapies may serve as a proxy for RA disease activity and severity. To date, the relationship between RA and dementia, as well as the association between RA and cognitive decline in dementia remain not fully understood and has not been widely studied.

In this study, we aimed to explore whether RA is associated with increased progression of cognitive decline and higher risk of all-cause mortality in patients with dementia.

## Methods

### Study Design and Data Source

Based on nationwide Swedish registers, we conducted a registry-based cohort study between May 1, 2007, and October 16, 2018, to examine the association between RA and cognitive decline in patients with dementia. Using the Swedish Registry for Cognitive/Dementia Disorders (SveDem), we identified patients with incident diagnosis of dementia. SveDem is a quality registry established in 2007 aiming to enhance dementia care by registering all patients with incident dementia in Sweden and ensuring annual follow-ups to track their diagnosis, treatment, and care over time.^20^ The registry covers the entire care chain, integrating data from 4 types of care units: specialist units, primary care, special housing, and home health care. Care units report data through a web-based system,^21^ enabling ongoing monitoring and comparison of care quality at local, regional, and national levels. SveDem includes patients with incident dementia diagnoses from either primary care or specialist memory clinics. The registry contains information on diagnostic variables, such as type of dementia, cognitive evaluation by Mini-Mental State Examination (MMSE) scores, coresident status, living in nursing home, and type of diagnostic unit. These variables are used in this study.

The Swedish National Patient Register contains nationwide records on inpatient care since 1987 and more than 80% of specialized (hospital-based) outpatient care since 2001 with high validity (85%–95%).^22^ The validity of the RA diagnosis in the Swedish National Patient Register was as high as 91%.^23^ The Swedish Prescribed Drug Register provides complete data on dispensation of prescription medications from all pharmacies since July 2005.^24^ Cause of Death Registry contains data on overall and specific mortality and dates of death, covering 100% of all deaths.^25^ In Sweden, the National Board of Health and Welfare links data across registers using the personal identification number. After linkage, the data are pseudonymized by assigning a unique identify number, which is then provided to researchers. In this study, a pseudonymized unique number was used to identify patients across sources and to merge data.

### Standard Protocol Approvals, Registrations, and Patient Consents

The requirement of written consent for this study was waived due to the register data being pseudonymized before delivery to our research group. The regional ethics committee in Stockholm approved the study, which complied with the Declaration of Helsinki. Participants and their caretakers were informed verbally and in writing about SveDem and had the option to decline participation.

### Study Population

The study population consisted of patients with dementia registered in SveDem, and followed up annually. We defined the index date as the date of the first recorded diagnosis of dementia. We excluded patients with missing information on MMSE score at baseline. In addition, we excluded patients diagnosed with rheumatic diseases (RD) other than RA before the diagnosis of dementia in both the RA and non-RA groups. This exclusion was implemented because of the distinct pathophysiologic differences between RA and other RDs.^26^ Finally, we excluded patients diagnosed with RD after dementia diagnosis in the non-RA group to ensure pure controls. eFigure 1 presents a flowchart for the selection of patients.

For dementia identified from SveDem, dementia diagnoses were coded as AD, mixed AD dementia, VaD, dementia with Lewy bodies (DLB), frontotemporal dementia (FTD), Parkinson disease dementia (PDD), and other dementias, which included unknown diagnosis and other dementia types not classified above. Dementia disorders are clinically diagnosed and recorded according to the International Classification of Diseases, Tenth Revision (ICD-10),^27^ with the McKeith criteria^28^ used for DLB, the Lund-Manchester criteria^29^ for FTD, and the Movement Disorder Society Task Force criteria^30^ for PDD, respectively. DLB and PDD were merged for this study as Lewy body dementias (LBDs) because they share pathologic and clinical characteristics, and many authors consider them part of a continuum within the spectrum of LBD.^31^

### Study Exposure

We used the ICD-10 codes from the National Patient Register to identify any first inpatient or outpatient records of RA before dementia diagnosis (eTable 1). We categorized the duration of exposure to RA before dementia diagnosis into 3 groups (<5, 5–10, and ≥10 years). We divided patients with RA into 4 groups (none, glucocorticoid, DMARDs, both glucocorticoid and DMARDs) based on the types of prescribed RA-related drugs, which was used as a proxy for RA disease activity and severity (eTable 2).

### Study Outcomes

The main outcome for this study was cognitive decline, defined as MMSE score change over the years. We collected information of baseline and follow-up MMSE scores from SveDem. The secondary outcome was all-cause death. The patients were observed from dementia diagnosis until the event of death, or end of follow-up (October 16, 2018), whichever occurred first. We extracted information on death causes from the Cause of Death Registry based on ICD-10 codes (eTable 3).

### Covariates

We defined covariates at the date of study entry, including age, sex, whether the patient was living alone, or living in nursing home, diagnosed in specialist care, and calendar year of dementia diagnosis. We used the ICD-10 codes to identify common and major comorbidities diagnosed before the date of dementia diagnosis (eTable 1). We used the Charlson Comorbidity Index (CCI) score^32^ (0, 1, 2, and ≥3, with higher values indicating more comorbidities) to assess medical comorbidities, using a weighted sum of diagnosed chronic disorders^33^ but excluding dementia. Data on ongoing medications were ascertained by the Anatomical Therapeutic Chemical codes and defined as the presence of filled pharmacy prescriptions within the 6 months before and at the date of dementia diagnosis (eTable 2). Cholinesterase inhibitors (ChEIs) and memantine were defined within 3 months after baseline and also at the date of each follow-up because they are antidementia medication (eTable 2).

### Statistical Analysis

We performed 1:3 propensity score matching without replacement using the nearest number matching method and with a caliper of 0.01 to balance confounders between patients with and without RA. We estimated the propensity score for being diagnosed with RA using logistic regression models based on age, sex, baseline MMSE score, dementia subtypes, living alone, diagnosis in specialist care, nursing home residency, comorbidities, and ongoing medications. The balance of baseline characteristics before and after weighting is shown in eFigure 2.

Continuous variables are presented as the mean with SD. Categorical variables are presented as percentage. We first compared the characteristics between individuals with RA and those without RA. We used mixed-effects repeated-measures models to examine the associations between RA and cognitive decline, measured by MMSE score changes over time. The beta coefficients (β) and 95% CIs were calculated with adjustments for RA (yes/no), follow-up time (year), and an interaction between RA and follow-up time. Follow-up time was treated as a continuous measure defined as years since first cognitive assessment. Patients were classified as dropout if they either died or had no follow-up MMSE recorded within 1 year after their last recorded MMSE. Reasons for missing data in surviving patients may include severe cognitive impairment making MMSE testing unfeasible, or loss of follow-up contact with secondary care. In addition, since patients with dementia tend to be older and have more comorbidities, they were at increased risk of mortality before the end of follow-up. To account for the potential effect of dropout or to the presence of a competing risk such as death, we calculated the inverse probability of censoring weighting to our analyses.^34^ Follow-up time had a strong nonlinear (approximately quadratic) relation with MMSE score (*p* < 0.05), so we also included a quadratic term of follow-up time in the adjusted model to consider a nonlinear trend in MMSE score across time. The goodness of fit of the models was assessed and compared by the Bayesian Information Criterion (BIC), with lower BIC value indicating a better fit, taking into account the model complexity. The final model included the quadratic terms of follow-up time, RA, and the interaction between RA and follow-up time. Next, we used the same strategy to investigate the association of different exposure time of RA before dementia diagnosis (<5, 5–10, and ≥10 years before dementia diagnosis) and RA-related drugs (no, glucocorticoid, DMARDs, both glucocorticoid and DMARDs). We tested the *p* trend for each class.

We estimated crude incidence rates per 1,000 person-years for all-cause death. We used Cox proportional hazards models to estimate the associations between RA and all-cause mortality, calculating the hazard ratios (HRs) with 95% CIs. Time since index date served as the underlying timescale. We compared the underlying causes of death between patients with RA and non-RA, in the whole propensity score matched cohort as well as in patients with AD and mixed AD dementia.

We conducted subgroup analyses to explore the potential effects of sex, age (<75, 75–84, ≥85 years), living status (alone/not alone), specialist care (no/yes), baseline MMSE (<20/≥20), CCI score (≤2/≥3), and the use of ChEIs (no/yes, among patients diagnosed with AD and mixed AD dementia), as well as memantine (no/yes, among patients diagnosed with AD and mixed AD dementia), on the association between RA and outcomes.

In addition, we evaluated sensitivity analyses to test the robustness of the results: (1) to account for floor effects, we conducted analyses on patients with an MMSE score ≥10, excluding those with severe dementia at baseline. (2) Because AD and mixed AD dementia account for more than 50% of the dementia population, we also conducted analyses on patients with AD and mixed AD dementia.

Statistical analyses were conducted with SAS version 9.4 (SAS Institute Inc, Cary, NC) and R 4.2.1, with statistical tests using a 2-tailed *p* < 0.05 as the level of statistical significance.

### Data Availability

Owing to Swedish regulations, the data in this study cannot be shared by the authors. However, the data can be requested from the 2 government agencies: The National Board of Health and Welfare (socialstyrelsen@socialstyrelsen.se) and Statistics Sweden (scb@scb.se).

## Results

### Patient Characteristics

The baseline demographic and clinical characteristics by RA in patients before and after matching are given in Table 1 and eTable 4. Patients with RA, compared with those without RA, were more likely to be female (73.4% vs 57.8%), be in nursing home (9.6% vs 8.2%), living alone (47.5% vs 44.8%), had more comorbidities, and took more medications at dementia diagnosis (eTable 4).

**Table 1:** Baseline Demographic and Clinical Characteristics of Patients With Dementia Stratified by RA in the Propensity Score Matched Cohort, 2007–2018

Variables	Non-RA (n = 5,055)	RA (n = 1,685)	*p* Value
Age at dementia diagnosis, mean ± SD, y	80.1 ± 7.5	79.9 ± 6.7	0.54
Sex, n (%)			0.85
Men	1,359(26.9)	449(26.6)	
Women	3,696(73.1)	1,236(73.4)	
Dementia diagnosis, n (%)			0.99
AD	1,510(29.9)	499(29.6)	
Mixed AD dementia	1,149(22.7)	379(22.5)	
Vascular dementia	1,110(22.0)	361(21.4)	
Lewy body disease	77(1.5)	26(1.5)	
Frontotemporal dementia	50(1.0)	18(1.1)	
Parkinson disease dementia	46(0.9)	19(1.1)	
Unspecified dementia	999(19.8)	344(20.4)	
Other diagnosis	114(2.3)	39(2.3)	
MMSE score, Mean ± SD	20.7 ± 5.0	20.7 ± 4.8	0.78
Nursing home, n (%)	478(9.5)	161(9.6)	0.91
Coresident status, n (%)			0.98
Living alone	2,402(47.5)	800(47.5)	
Cohabiting	2,653(52.5)	885(52.5)	
Diagnostic unit, n (%)			0.78
Specialist care	2,831(56.0)	937(55.6)	
Primary care	2,224 (44.0)	748 (44.4)	
Calendar y, n (%)			0.26
2007	59(1.2)	15(0.9)	
2008	168(3.3)	46(2.7)	
2009	288(5.7)	68(4.0)	
2010	364(7.2)	123(7.3)	
2011	451(8.9)	139(8.2)	
2012	513(10.1)	171(10.1)	
2013	558(11.0)	182(10.8)	
2014	634(12.5)	220(13.1)	
2015	628(12.4)	223(13.2)	
2016	563(11.1)	206(12.2)	
2017	507(10.0)	188(11.2)	
2018	322(6.4)	104(6.2)	
Comorbidities, n (%)			
Diabetes mellitus	1,014(20.1)	341(20.2)	0.88
Hypertension	2,766(54.7)	917(54.4)	0.83
Congestive heart failure	827(16.4)	278(16.5)	0.89
Myocardial infarction	745(14.7)	250(14.8)	0.92
Peripheral vascular disease	391(7.7)	125(7.4)	0.67
Cerebrovascular disease	1,141(22.6)	382(22.7)	0.93
Chronic obstructive pulmonary disease	827(16.4)	261(15.5)	0.40
Chronic kidney disease	290(5.7)	91(5.4)	0.61
Liver disease	81(1.6)	30(1.8)	0.62
Cancer	2,444(48.3)	823(48.8)	0.73
Stroke	811(16.0)	275(16.3)	0.79
Atrial fibrillation	1,091(21.6)	356(21.1)	0.69
Alcohol abuse	117(2.3)	38(2.3)	0.89
Fracture	1962(38.8)	669(39.7)	0.52
Depression	533(10.5)	178(10.6)	0.98
Hearing loss	664(13.1)	226(13.4)	0.71
Medication use, n (%)			
ACEi/ARBs	2,137(42.3)	701(41.6)	0.63
B-blocking agents	2,202(43.6)	750(44.5)	0.50
Calcium channel blockers	1,243(24.6)	417(24.7)	0.90
Lipid modifying agents	1,693(33.5)	560(33.2)	0.85
NSAIDs	1,064(21.0)	358(21.2)	0.86
Glucocorticoid	2,307(45.6)	775(46.0)	0.80
DMARDs			
csDMARDs	—	756 (44.9)	—
tsDMARDs/bDMARDs	—	74 (4.4)	—
Antithrombotic agents	4,154(82.2)	1,386(82.3)	0.94
ChEIs^a^	775 (29.2)	269 (30.6)	0.40
Memantine^a^	169 (5.5)	53 (6.0)	0.54
Antidepressants	1834(36.3)	600(35.6)	0.62
Anxiolytics	1,013(20.0)	327(19.4)	0.57
Hypnotics	1,600(31.7)	545(32.3)	0.60
Antipsychotics	323(6.4)	119(7.1)	0.33

Abbreviations: ACEi = angiotensin-converting enzyme inhibitor; AD = Alzheimer dementia; ARB = angiotensin receptor blocker; bDMARDs = biologic disease-modifying antirheumatic drugs; ChEIs = cholinesterase inhibitors; csDMARDs = conventional synthetic disease-modifying antirheumatic drugs; DMARDs = disease-modifying antirheumatic drugs; MMSE = Mini-Mental State Examination; NSAID = nonsteroidal anti-inflammatory drug; RA = rheumatoid arthritis; SD = standard error; tsDMARDs = targeted synthetic disease-modifying antirheumatic drugs.

Continuous variables are reported as mean ± SD, and categorical variables are reported as n (%).

a Among patients diagnosed with AD and mixed AD dementia.

The final propensity score-matched cohort included 1,685 patients with RA (mean [SD] age, 79.9 (6.7) years; 73.4% were women) and 5,055 non-RA (mean [SD] age, 80.1 (7.5) years; 73.1% were women). The median follow-up was 2.9 years (interquartile range, 1.5–4.6 years) for non-RA and 2.6 years (interquartile range, 1.4–4.2 years) for RA (Table 1). The 2 groups were well balanced after matching, which removed many of the significant imbalances between 2 groups, especially in sex, comorbidities, and ongoing medications (eFigure 2).

### RA and Cognitive Decline

In total, 111,266 MMSE measurements were available for analysis. The dropout rate was 63.7%, with 3,391 patients who died and 900 patients who survived but had no further MMSE data available. At baseline, the mean (SD) MMSE score was 20.7 (4.8) in RA and 20.7 (5.0) in non-RA. As given in Table 2, compared with non-RA patients, RA was significantly associated with faster cognitive decline (β = −0.24 points/y; 95% CI −0.38 to −0.10; *p* = 0.001). We found significant trends with RA disease exposure time (*p* = 0.001) and RA-related drugs (*p* < 0.001) for cognitive decline (Table 2). Compared with non-RA patients, longer exposure time for RA (5–10 years, β = −0.29 points/y; 95% CI −0.47 to −0.11; *p* = 0.001) and prescription of DMARDs with (β = −0.36 points/y; 95% CI −0.69 to −0.03; *p* = 0.03) or without (β = −0.38 points/y; 95% CI −0.59 to −0.18; *p* < 0.001) glucocorticoids were associated with faster cognitive decline during follow-up (Table 2). The associations between RA and cognitive decline were consistent in sensitivity analysis when excluding patients with baseline severe dementia (eTable 5) and in patients with AD and mixed AD dementia (eTable 6).

**Table 2:** Associations Between RA and Cognitive Decline in Propensity Score Matched Cohort Study

Variables	Coefficient (95% CI)^a^	*p* Value
Non-RA (n = 5,055)	Ref	
RA (n = 1,685)	−0.24 (−0.38 to −0.10)	0.001
RA exposure time		
Non-RA (n = 5,055)	Ref	
<5 y (n = 451)	−0.11 (−0.43 to 0.22)	0.52
5–10 y (n = 592)	−0.29 (−0.47 to −0.11)	0.001
≥10 y (n = 642)	−0.18 (−0.47 to 0.09)	0.17
*p* for trend		0.001
RA-related drugs		
Non-RA (n = 5,055)	Ref	
No RA-related drugs (n = 571)	0.02 (−0.23 to 0.26)	0.90
Glucocorticoid (n = 332)	−0.16 (−0.54 to 0.23)	0.43
DMARDs (n = 339)	−0.38 (−0.59 to −0.18)	<0.001
Glucocorticoid + DMARDs (n = 443)	−0.36 (−0.69 to −0.03)	0.03
*p* for trend		<0.001

Abbreviations: DMARDs = disease-modifying antirheumatic drugs; RA = rheumatoid arthritis.

a β (95% CI) is obtained from 1:3 propensity score matched cohort.

**Figure 1:**
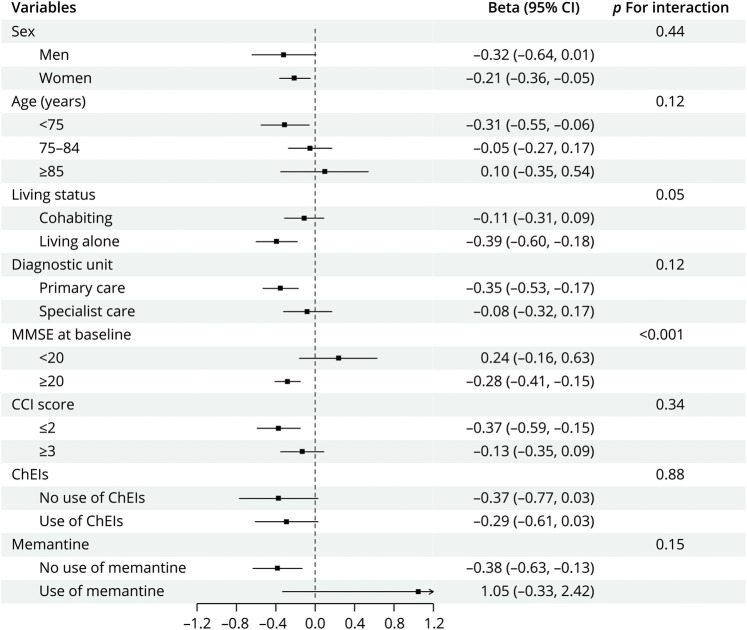
Subgroup Analyses of the Association Between RA and Cognitive Decline in Propensity Score Matched Cohort, 2007–2018 *Analyses were stratified by ChEI (no/yes) and memantine (no/yes) use among patients diagnosed with AD and mixed AD dementia, n = 3,537. AD = Alzheimer dementia; CCI = Charlson Comorbidity Index; ChEIs = cholinesterase inhibitors; HR = hazard ratio; MMSE = Mini-Mental State Examination; RA = rheumatoid arthritis.

Subgroup analysis revealed consistency in our findings across different age groups (75, 75–84, ≥85 years), sex, places of diagnosis (primary care or specialist care), CCI (≤2 or ≥3), and use of antidementia medications (ChEIs or memantine) (Figure 1). RA was notably associated with cognitive decline in individuals with higher baseline MMSE values (≥20) (β = −0.28 points/y; 95% CI −0.41 to −0.15; *p* < 0.001) and those living alone (β = −0.39 points/y; 95% CI −0.60 to −0.18; *p* < 0.001). Significant interactions were observed between RA, baseline MMSE scores, and living conditions regarding cognitive decline (*p* for interaction <0.05).

### RA and All-Cause Death

**Figure 2:**
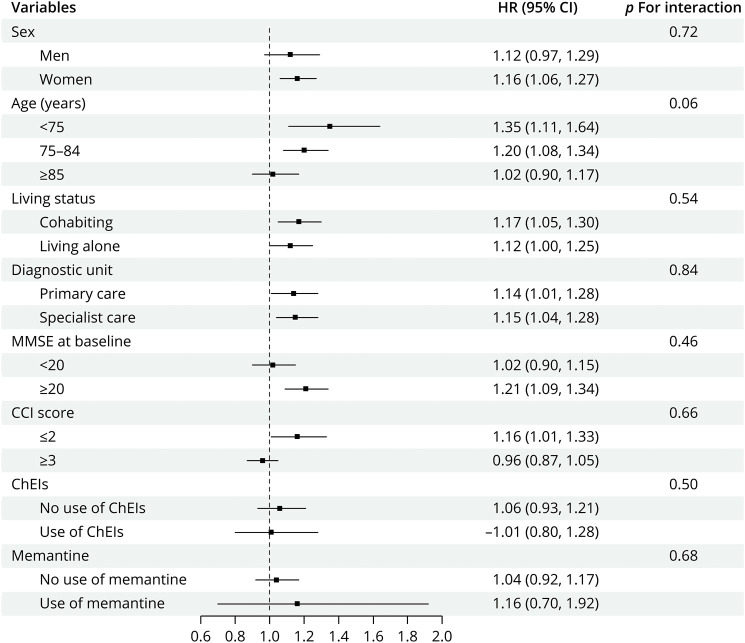
Subgroup Analyses of the Associations Between RA and All-Cause Mortality in Propensity Scored Matched Cohort, 2007–2018 *Analyses were stratified by ChEI (no/yes) and memantine (no/yes) use among patients diagnosed with AD and mixed AD dementia, n = 3,537. AD = Alzheimer dementia; CCI = Charlson Comorbidity Index; ChEIs = cholinesterase inhibitors; HR = hazard ratio; MMSE = Mini-Mental State Examination; RA = rheumatoid arthritis.

During a median 2.8 (IQR: 1.4–4.5) years of follow-up, corresponding to 21,520 person-years, 3,391 (50.3%) patients died. A higher mortality rate was observed for patients with RA (170.30 per 1,000 py; 95% CI 158.96–181.64) vs non-RA (153.64 per 1,000 py; 95% CI, 147.64–159.63). The most common underlying causes of death in the whole propensity score matched cohort or in patients with AD and mixed AD dementia were neurologic disease and cardiovascular disease, followed by cancer and respiratory diseases (eTable 7). Compared with non-RA patients, patients with RA had an increased risk of death (HR = 1.15, 95% CI 1.06–1.24, *p* = 0.001) (Table 3 and eFigure 3). The analysis by RA exposure time and RA-related drugs showed that longer RA disease exposure time (5–10 years, HR = 1.13; 95% CI 1.01–1.27, *p* = 0.03; ≥10 years, HR = 1.21; 95% CI 1.07–1.37, *p* = 0.01) and prescription of glucocorticoids with (HR = 1.28; 95% CI 1.12–1.46, *p* < 0.001) or without (HR = 1.47; 95% CI 1.27–1.70, *p* < 0.001) DMARDs were associated with increased risk of all-cause mortality, compared with non-RA patients (Table 3). These associations were consistent throughout sensitivity analyses when estimating from patients with nonsevere dementia but lost the association in patients with AD and mixed AD dementia (eTables 8 and 9). We observed a similar trend across subgroup analyses (Figure 2).

**Table 3:** Associations Between RA and All-Cause Mortality in Propensity Score Matched Cohort Study

Variables	Events	Person-time, y	Incidence rate per 1,000 py^a^	HR (95% CI)^b^	*p* Value
Non-RA (n = 5,055)	2,525	16,434.9	153.64 (147.64–159.63)	Ref	
RA (n = 1,685)	866	5,085.2	170.30 (158.96–181.64)	1.15 (1.06–1.24)	0.001
RA exposure time					
Non-RA (n = 5,055)	2,525	16,434.9	153.64 (147.64–159.63)	Ref	
<5 y (n = 451)	245	1,465.8	167.14 (146.21–188.07)	1.10 (0.97–1.25)	0.16
5–10 y (n = 592)	335	1940.7	172.62 (154.13–191.10)	1.13 (1.01–1.27)	0.03
≥10 y (n = 642)	286	1,675.7	170.67 (150.89–190.46)	1.21 (1.07–1.37)	0.01
*p* for trend					<0.001
RA-related drugs					
Non-RA (n = 5,055)	2,525	16,434.9	153.64 (147.64–159.63)	Ref	
No RA-related drugs (n = 571)	286	1803.4	158.59 (140.21–176.97)	1.06 (0.94–1.20)	0.37
Glucocorticoid (n = 332)	195	912.3	213.75 (183.74–243.75)	1.47 (1.27–1.70)	<0.001
DMARDs (n = 339)	146	1,092.1	133.69 (112.00–155.37)	0.89 (0.75–1.05)	0.16
Glucocorticoid + DMARDs (n = 443)	239	1,274.4	187.54 (163.76–211.32)	1.28 (1.12–1.46)	<0.001
*p* for trend					0.001

Abbreviations: DMARDs = disease-modifying antirheumatic drugs; HR = hazard ratio; RA = rheumatoid arthritis.

a Incidence rate (unweighted cohort) is presented as number of events per 1,000 patient-years.

b Hazard ratio is obtained from 1:3 propensity score matched cohort.

## Discussion

In this longitudinal population-based registry study in patients with incident dementia, we found that compared with patients without RA, patients diagnosed with RA presented faster cognitive decline and had increased risk of mortality. Longer RA disease exposure time and prescription of DMARDs were associated with a faster cognitive decline during follow-up, while glucocorticoid use was significantly associated with increased mortality.

There is a growing body of evidence that long-term chronic systemic inflammation is associated with cerebral inflammation, despite the blood-brain barrier.^8,35^ The latter occurred alongside the presence of β-amyloid plaques,^36^ resulting in increased levels of inflammatory molecules and cytokines in the serum of patients with AD.^37^ In addition, systemic inflammation is associated with cerebral microvasculature disease in VaD.^10^ Neuroinflammation has been shown across different phases of dementia pathology. RA is the most common form of inflammatory arthritis, and concomitant systemic inflammation may accelerate the progression of dementia, making patients with RA a model population for studying how chronic systemic inflammation affects cognitive progression in patients with dementia after diagnosis.^38^

Previous evidence has indicated that RA was associated with increased risk of AD and other dementias.^17^ A recent meta-analysis has demonstrated a higher risk of dementia in patients presenting with RA, chronic inflammation, and high CRP in midlife.^39^ However, the relationship between RA and cognitive decline in patients with dementia has not been clearly demonstrated. In this study, we presented novel findings that RA was associated with adverse cognitive outcomes in all patients with dementia and in patients with AD and mixed AD dementia. This study represents a population-based, propensity score-matched analysis investigating the potential association between RA and cognitive decline in patients with incident dementia. We also observed that cognitive function was more severely affected during follow-up in dementia patients with longer exposure duration for RA. The findings were consistent with a previous population-based cohort study that observed the risk of dementia in individuals with RA and its associated disease characteristics and activity.^40^ New treatment strategies have substantially changed the course of RA over the past few decades, involving treat-to-target strategies, combination therapy, and use of bDMARDs and tsDMARDs. Thus, while our study focuses on the prognosis of patients with both dementia and RA, the broader link between chronic systemic inflammation and dementia pathogenesis might be supported by previous findings of a lower incidence of dementia in RA in recent years,^41^ potentially due to early recognition of RA and improved treatment strategies that aim for early and sustained remission or low disease activity. Indeed, it seems that the risk of dementia is declining over time, compared with non-RA referents, and individuals with RA diagnosed in 2000s had insignificantly lower cumulative incidence of dementia than those in the 1980s.^41^

RA is typically treated initially with short-term glucocorticoid and long-term csDMARD therapy.^19^ The evidence on association between the use of RA medications and the risk of dementia is somewhat contradictory. More recently, observational studies have hinted at how DMARDs and tumor necrosis factor–inhibiting biological therapies could reduce the incidence of dementia,^42^ but subsequent interventional studies have been disappointing.^43^ Furthermore, research on whether therapies designed to treat inflammatory and autoimmune diseases have the potential to slow down cognitive decline in dementia is lacking. In this study, most patients with RA (44.9%) were prescribed csDMARDs (44.9%) and glucocorticoids (46%), and only a few of them were prescribed tsDMARDs or bDMARDs (4.4%). We found that prescription of DMARDs, but not glucocorticoids alone before dementia diagnosis, was associated with faster cognitive decline compared with non-RA patients. Although no clinical data on disease activity were available in this analysis, this finding may support a link between chronic inflammation and worse cognition. A propensity score-matched case-control study^44^ using Taiwan National Health Insurance data showed an increased risk for dementia (specifically, VaD) among patients with RA who used csDMARDs, but not among those who used bDMARDs or NSAIDs. Since there was limited prescription of bDMARDs and tsDMARDs in our study, it precludes comparisons between these 2 treatment modalities. Future interventional trials are needed to investigate whether therapies designed to treat inflammatory and autoimmune diseases have the potential to be used to treat dementia.

ChEIs are approved pharmacologic therapies with the potential to offset cognitive decline in persons with AD.^4^ ChEIs inhibit the acetylcholine (ACh)-degrading enzyme acetylcholinesterase, leading to increasing levels and duration of action of ACh in the synapses of both central and peripheral nervous systems.^45^ Besides its action as an acetylcholinesterase inhibitor, the anti-arthritic effect of galantamine, demonstrated by the reduction in all biomarkers of inflammation, has been observed in previous studies.^46,47^ However, this study did not show a significant difference in the effect of RA on cognitive decline between patients under treatment with ChEIs or not. So far, the interactive effects of RA and ChEIs on cognition in patients with dementia are lacking. The possibility of confounding by indication/contraindication and channelling bias should be considered. Investigating other ChEIs in combination with anti-rheumatic treatments in incident RA and dementia with long follow-up could help to better understand the relevance of our observation.

In this study, compared with non-RA patients, patients with RA diagnosed with dementia had a significantly higher risk of all-cause death, especially for those who had longer exposure duration for RA and for those who were prescribed glucocorticoids. Glucocorticoid treatment has been reported to be associated with dementia in patients with RD such as dermatomyositis and polymyositis.^48^ A German prospective cohort study found that patients with long-term RA disease activity faced a substantially increased risk of death, with higher doses of glucocorticoids associated with a further increased risk.^49^ Furthermore, a population-based study of 604 patients with RA showed increased mortality in patients treated with low-dose oral glucocorticoids for more than 10 years compared with patients who did not receive glucocorticoids or who were treated for less than 10 years.^50^ This finding may be related to long-term exposure to systemic inflammation, and prolonged treatment with glucocorticoids in RA populations,^49,50^ but in our study, the observed higher risk of all-cause death in RA patients with dementia seems less likely to be related to side effects of glucocorticoids per se because of similar prescription of glucocorticoids in patients with RA and non-RA matched controls.

Strengths of this study include large sample size and the use of data from standard patient registration and thus reflect real-world data. This study also examined the effect of RA disease on cognitive function in the long-term follow-up in patients with dementia in comparison with those without RA. In addition, the duration of RA before dementia diagnosis, prescription of RA-related medications, and different dementia disorders were considered.

Our study has some limitations. First, although we used a propensity score-matched cohort design to control for unbalanced confounders in our study, we cannot rule out the possibility of unmeasured confounders. Second, since RA was extracted from the national registers, the duration of disease before dementia onset could not be evaluated. Furthermore, information on RA severity is not available in our data set. While the use of combination therapies may serve as a proxy for RA disease activity and severity, we cannot rule out the possibility that the results are influenced by the effects of the medication itself rather than the severity of the disease, and vice versa. Third, the assessment of the study's exposure is based on clinical diagnoses obtained through specialized inpatient and outpatient care, primarily representing more severe RA cases. Furthermore, our cognitive functioning data were collected in real-world clinical practice, so the number of MMSE measurements performed was different between individuals, and the missing of MMSE measurements was not at random and attrition to follow-up was high, at 63.7%; however, we attempted to address the potential concern of missing of follow-up by adjusting the estimates using inverse probability of censoring weighting. Finally, while our study used MMSE to evaluate cognitive function in patients with dementia, there are some inherent limitations associated with this measure. The MMSE is known to have a floor effect, which means it may not be sensitive enough to detect changes in very severe cognitive impairment. However, in SveDem, the MMSE follow-ups are mostly performed at least 12 months apart, which can be considered a sufficient time interval to reduce the risk for practice effects in individuals with dementia, although it cannot be fully ruled out. Future studies should consider using a broader range of cognitive assessments to capture different stages of cognitive impairment, more sensitive to change, and including alternative tests that are less vulnerability to floor effects and practice effects.

In this registry-based study, we found that RA was associated with cognitive worsening in patients with dementia and an increased risk of mortality compared with non-RA dementia patients. Our findings highlight the importance of appropriate management and treatment for dementia patients with RA.

## Glossary

AD: Alzheimer disease
BIC: Bayesian Information Criterion
CCI: Charlson Comorbidity Index
ChEI: cholinesterase inhibitor
csDMARD: commonly treated with conventional DMARD
DLB: dementia with Lewy body
DMARD: disease-modifying antirheumatic drug
FTD: frontotemporal dementia
HR: hazard ratio
ICD-10: International Classification of Diseases, tenth revision
LBD: Lewy body dementia
MMSE: Mini-Mental State Examination
PDD: Parkinson disease dementia
RA: rheumatoid arthritis
RD: rheumatic disease
tsDMARD: targeted synthetic DMARD
VaD: vascular dementia
